# Molecular mechanism of acquired drug resistance in the EGFR‐TKI resistant cell line HCC827‐TR

**DOI:** 10.1111/1759-7714.13342

**Published:** 2020-03-12

**Authors:** Tao Yu, Qian Xia, Ting Gong, Jing Wang, DianSheng Zhong

**Affiliations:** ^1^ Department of Medical Oncology Tianjin Medical University General Hospital Tianjin China; ^2^ Tianjin Medical University Tianjin China; ^3^ Department of Lung Cancer Institute Tianjin Medical University General Hospital Tianjin China

**Keywords:** Acquired resistance, EGFR‐TKIs, FGF2, non‐small cell lung cancer

## Abstract

**Background:**

The first‐line standard treatment of non‐small cell lung cancer (NSCLC) with *EGFR* mutation is EGFR‐tyrosine kinase inhibitors (TKIs). However, most patients will develop acquired resistance after 9–13 months. This study investigated novel molecular mechanisms of acquired resistance to EGFR‐TKIs to identify a potential new treatment for EGFR‐TKI resistant NSCLC patients.

**Methods:**

We established an EGFR‐TKI resistant cell line (HCC827‐TR) by culturing the HCC827‐P cell line through continuous erlotinib culture. We used Sanger sequencing, RT‐PCR, and western blot to rule out known resistance mechanisms in HCC827‐TR cells, including EGFR‐T790M and *MET*, *PTEN,* or *EGFR* expression changes. Next‐generation sequencing was performed and identified differentially expressed genes between two cell lines and examined the genes with GO and KEGG pathway database analyses. We also examined the molecular alterations in COSMIC and GDSC databases and performed hazard predictions using SIFT, PolyPhen‐2, Mutation Taster, and CADD.

**Results:**

Our results identified *FGF2* as a differentially expressed gene with a G101T point mutation in HCC827‐TR cells that showed high mutation frequency and hazard score. HCC827‐TR cells showed elevated FGF2 compared to parental cells. It is noteworthy that treatment with the FGFR inhibitor AZD4547 could restore the sensitivity of HCC872‐TR cells to erlotinib.

**Conclusions:**

An erlotinib‐resistant cell line HCC827‐TR was successfully constructed and we identified the EGFR‐TKI resistance mechanism involving the *FGF2* gene mutation. Targeted inhibition of the FGF2/FGFR signaling pathway may effectively restore the sensitivity of the resistant cells to erlotinib. These results suggest a novel treatment strategy for EGFR‐TKI resistant NSCLC patients.

**Key points:**

- **Significant findings of the study**: Identifies a novel molecular mechanism for EGFR‐TKI acquired resistance.
- **What this study adds**: A potential novel strategy for the treatment of EGFR‐TKI resistant NSCLC patients.

## Introduction

Non‐small cell lung cancer (NSCLC) accounts for approximately 80% of all lung cancer cases,^1^ and a large proportion of NSCLC cases are already in the advanced stages at the time of diagnosis. For many decades, the standard treatment of NSCLC was double‐drug chemotherapy with platinum; however, the five‐year survival rate was only 15%.^2^ In the early 2000s, the approval of gefitinib, a first generation epidermal growth factor receptor tyrosine kinase inhibitor, led the way for molecular targeted therapy in lung cancer. Currently, many other targeted drugs have been approved for clinical treatment. These driver mutations include ALK translocations,^3^ BRAF V600E mutation,^4^ MET exon 14 skipping,^5^ ROS1^6^ and RET rearrangement.^7^

*EGFR* mutation is the most common genetic variant (50%–60%) in lung adenocarcinoma patients in East Asia. The IPASS study^8^ first found that *EGFR* mutation is an important strong predictor of the clinical efficacy of EGFR‐TKIs in lung adenocarcinoma. The deletion of exon 19 and the L858R point mutation in exon 21 are the most common *EGFR* mutation types and confer sensitivity to EGFR‐TKIs. Several large randomized phase III clinical trials, such as First‐SIGNA,^9^ WJTOG3405,^10^ NEJ002,^11^ OPTIMAL,^12^ ENSURE,^13^ and EURTAC,^14^ demonstrated that the curative effect of targeted therapy for lung adenocarcinoma with *EGFR* sensitive mutations is significantly better than that with traditional chemotherapy. Meanwhile, the side effects can be well controlled as compared to those of chemotherapy. Accordingly, EGFR‐TKI has become a standard first‐line treatment for advanced NSCLC with *EGFR‐*TKI‐sensitive mutations.

Despite the initial beneficial effects of EGFR‐TKI in patients with *EGFR* mutation, the overwhelming majority of patients acquire resistance after 9–13 months, leading to disease progression.^15^ The most important acquired resistance mechanism is the secondary *EGFR* mutation of T790M that occurs in exon 20 and accounts for 50%–60% of all cases.^16^ Previous studies have reported that *MET* amplification,^17^ the loss or decline of activating *EGFR* mutant gene,^18^ and *PTEN* deletion^19^ could also lead to EGFR‐TKI resistance. In addition, the transformation of tumor tissue types, epithelial mesenchymal change, epigenetic changes, and abnormal microenvironment of tumor cells may cause resistance to EGFR‐TKIs; the mechanisms for approximately 18%–20% of cases with acquired resistance remain unknown.

The purpose of this study was to first establish an EGFR‐TKI drug‐resistant cell line and screen for resistance mechanisms. We then conducted high throughput whole exon sequencing of the primary cell line and drug‐resistant cell line to identify and examine potential differentially expressed genes that might lead to drug resistance. We also performed targeted inhibition of the identified gene signal downstream pathway in tumor cells to observe changes of drug resistance to confirm the significance for further clinical study.

## Methods

### Cell lines and culture

HCC827‐P cells were obtained from an American type culture collection and cultured in RPMI‐1640 medium at 37°C in a saturated humidity atmosphere containing 5% CO_2_. The cells were subcultured when they reached 80%–90% adherence on the culture dishes and showed good morphology.

### Establishment of the drug‐resistant cell line

HCC827‐P cells were cultured in a medium containing erlotinib at 1 nmol/L. When the cells showed viability similar to that of cells without erlotinib, we gradually increased the concentration of erlotinib until it reached 1 μmol/L. The entire induction period lasted approximately eight months.

### MTS assays

Cells were plated into 96‐well plates at 200 μL cell suspension containing approximately 1.0 × 10^4^ cells in each well. After incubation at 37°C for 24 hours, different concentrations of erlotinib were added into the wells in triplicate. The half maximal inhibitory concentration (IC_50_) was calculated using curve regression analysis.

### Sanger sequencing

DNA was extracted from HCC827‐P and HCC827‐TR cell lines using the TRIzol Reagent Kit (Invitrogen, Carlsbad, California, US). Four dNTPs were added to the PCR reaction system. Fluorescence labeling was performed, and the samples were tested with urea‐modified polyacrylamide gel electrophoresis.

### Real time PCR (RT‐PCR)

Total RNA was extracted using the TRIzol Reagent Kit (Invitrogen, Carlsbad, California, USA); cDNA reverse transcription was performed using the Reverse Transcription Kit (Takara, Beijing, China) as per the manufacturer's instructions. RT‐PCR was performed using Quantitative PCR Kit (Takara, Beijing, China). The primer pairs are shown in Table S1. The expression of target genes was normalized to *GAPDH* using the 2^−ΔΔCt^ method.

### Western blot

Cells were lysed, and total protein was extracted. The proteins were separated with sodium dodecyl sulfate polyacrylamide gel electrophoresis and were then transferred to a PVDF membrane. After blocking, the membranes were incubated with primary antibodies (GAPDH (sc‐47 724) from Santacruz, Delaware Ave, USA; PTEN (9559L), FGF2 (3196S), FGFR (3471S), FRS2 (3864L) from cell signaling, Boston, USA) at 4°C overnight and then incubated with secondary antibody. Protein bands were analyzed using the Chemi Doc XRS System (Bio‐Rad, Hercules, CA, USA).

### Next‐generation sequencing (NGS)

Total DNA was extracted using the TRIzol Reagent Kit. Genomic DNA was randomly fragmented into DNA fragments of 180–250 bp. A DNA library was prepared, and liquid phase hybridized with biotin‐labeled probes. We captured the exons of target genes and performed single molecule amplification. The whole exon region of two cell lines were detected and contrasted with the Agilent‐V6 chip capture system in Illumina HiSeq2500 sequencing platform (150 bp paired end).

### Flow cytometry

Cells were plated in six‐well plates (2 × 10^5^ cells/well) and treated with various concentrations of erlotinib alone, or in combination with AZD4547 in triplicate. After being cultured and collected, they were added to an Eppendorf tube with 5 μL Lysol phosphatidyl ethanolamine (LPE, Rongbio, Shanghai, China), 5 μL 7‐amino‐actinomycin D (7‐AAD, Yeasen, Shanghai, China), and 400 μL loading buffer; the cells were analyzed for apoptotic populations using flow cytometry (FACSAriaTM, Becton Dickinson, USA).

### Statistical analysis

Statistical analyses were performed using SPSS 23.0 software. Data are shown as mean ± standard deviation values and analyzed with one‐way ANOVA or independent samples *t*‐tests to examine the difference among different treatment groups. A value of *P* < 0.05 was considered statistically significant.

## Results

### Construction of the HCC827‐TR drug‐resistant cell line

#### Cell morphology using microscopy and inhibition rate

**Figure 1:**
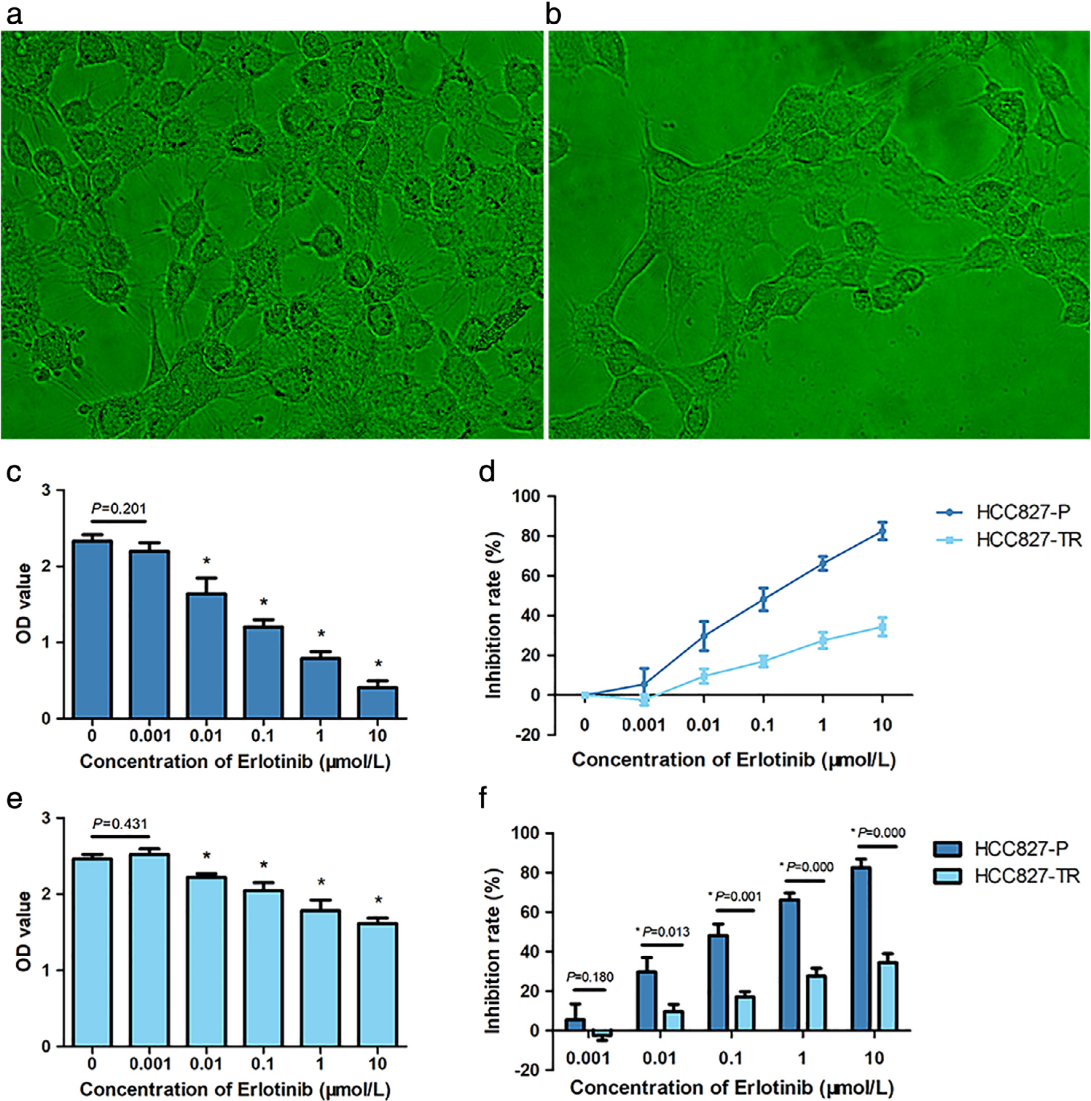
Induce and detect drug‐resistant cells: **(a)** Phase contrast microscopy of HCC827‐P cells and **(b)** HCC827‐TR cells (magnification, 100×). MTS assays of **(c)** HCC827‐P cells or **(d)** HCC827‐TR cells treated with erlotinib at concentrations of 0, 0.001, 0.01, 0.1, 1, or 10 μmol/L for 48 hours. **(e)** The concentration‐inhibition curve of erlotinib. **(f)** The comparison of inhibition rates at different erlotinib concentrations between HCC827‐P and HCC827‐TR cell lines.

We established an erlotinib‐resistant cell line (HCC827‐TR) using HCC827‐P as described in the Methods section. Phase contrast microscopy demonstrated that HCC827‐P and HCC827‐TR showed an epithelioid monolayer arrangement. HCC827‐P had clear boundaries and were arranged regular with round or oval shapes, a large nucleus and attached to each other (Fig 1(a), 100×). In contrast, the HCC827‐TR was arranged in a disorderly manner with an unclear boundary; the cells showed various shapes, such as long spindle, oval, and polygonal (Fig 1(b), 100×).

We next performed MTS assays in HCC827‐P; there were significant differences among 10 μmol/L and other groups (0.001, 0.01, 0.1, and 1 μmol/L, all *P* < 0.05) (Fig 1(c)). The concentration‐inhibition rate curve indicated that erlotinib inhibited the proliferation of HCC827‐P cells (IC_50_: 0.1 μmol/L) in a concentration‐dependent manner (Fig 1(e)). We did not detect any significant difference in the HCC827‐TR cells treated with 0.001 μmol/L erlotinib compared to controls (*P* = 0.431), but there was significant statistically differences between the other groups (all *P* < 0.05) (Fig. 1(d)). At the highest concentration of erlotinib, the inhibition rate was 34.5%. We found a significant difference in the inhibition rates between HCC827‐P and HCC827‐TR from 0.01 μmol/L to 10 μmol/L (all *P* < 0.02) (Fig 1(f)).

#### Screening of molecular factors involved in known drug resistance mechanisms

**Figure 2:**
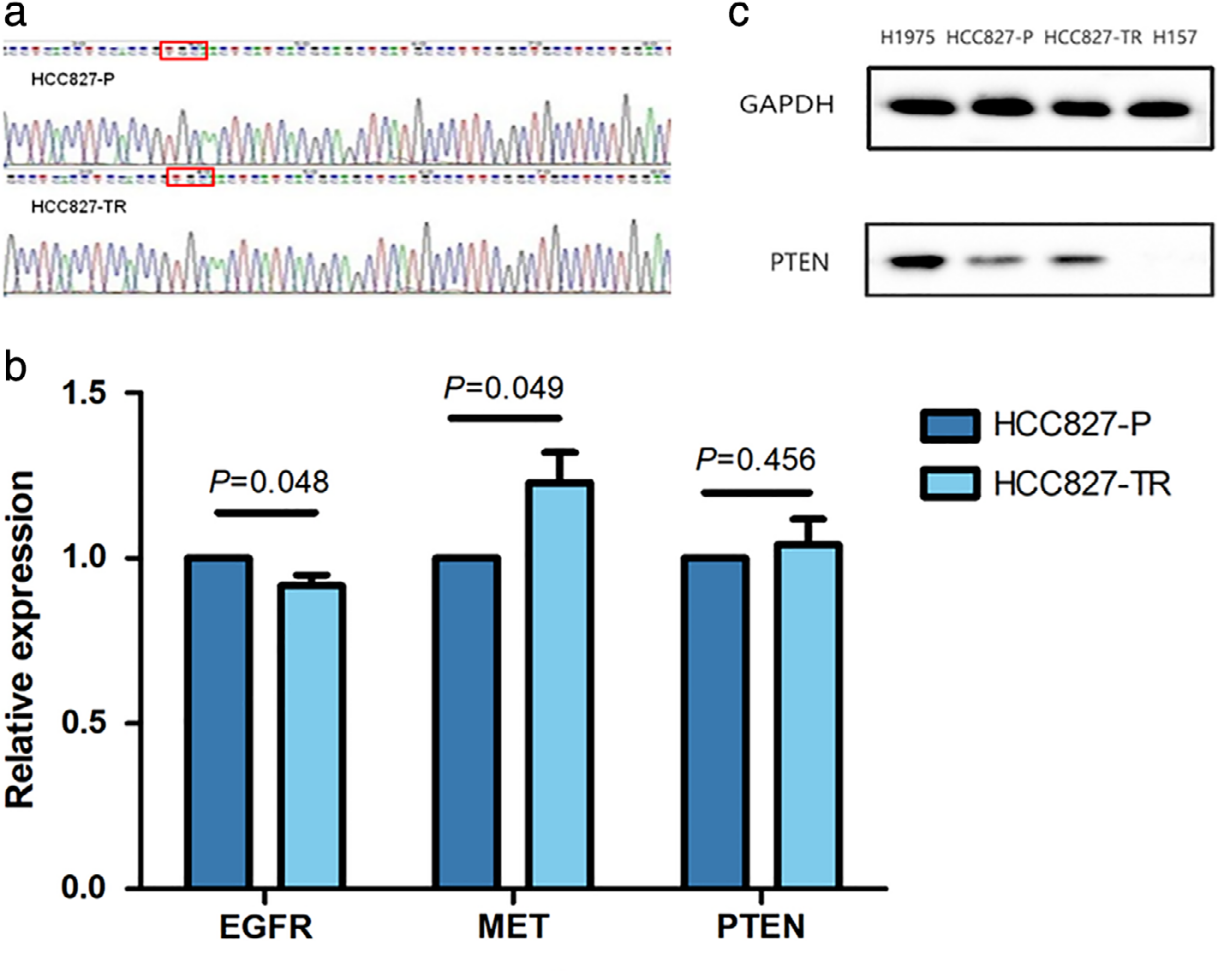
Screen known resistance genes: **(a)** Nucleotide sequencing results of EGFR in HCC827‐P and HCC827‐TR cell lines. **(b)** RT‐PCR analysis of *EGFR*, *MET*, and *PTEN* in HCC827‐P and HCC827‐TR cell lines. **(c)** Western blot analysis of PTEN protein in HCC827‐P, HCC827‐TR, H1975, and H175 cells.

Sanger sequencing revealed that an *EGFR* exon 19 deletion mutation from E746 to A750 was found in both cell lines; however, no changes were found at the 790 residue in *EGFR* in the HCC827‐TR (Fig 2(a)). Thereafter, we examined the expressions of EGFR (*P* = 0.048) and MET (P = 0.049); these were altered, but the difference was not significant. Similarly, the expressions of PTEN in the two cell lines also showed no statistical difference (P = 0.456) (Fig. 2(b)). We then examined PTEN protein in HCC827‐P and HCC827‐TR cell lines, along with H1975 and H157 which are *PTEN‐*positive lung cancer cell line and *PTEN‐*deficient lung cancer cell line, respectively. There was no significant difference between HCC827‐P and HCC827‐TR cells (Fig. 2(c)).

### Exploration of the drug resistance mechanism of HCC827‐TR cells

#### Somatic molecular alterations screening

**Figure 3:**
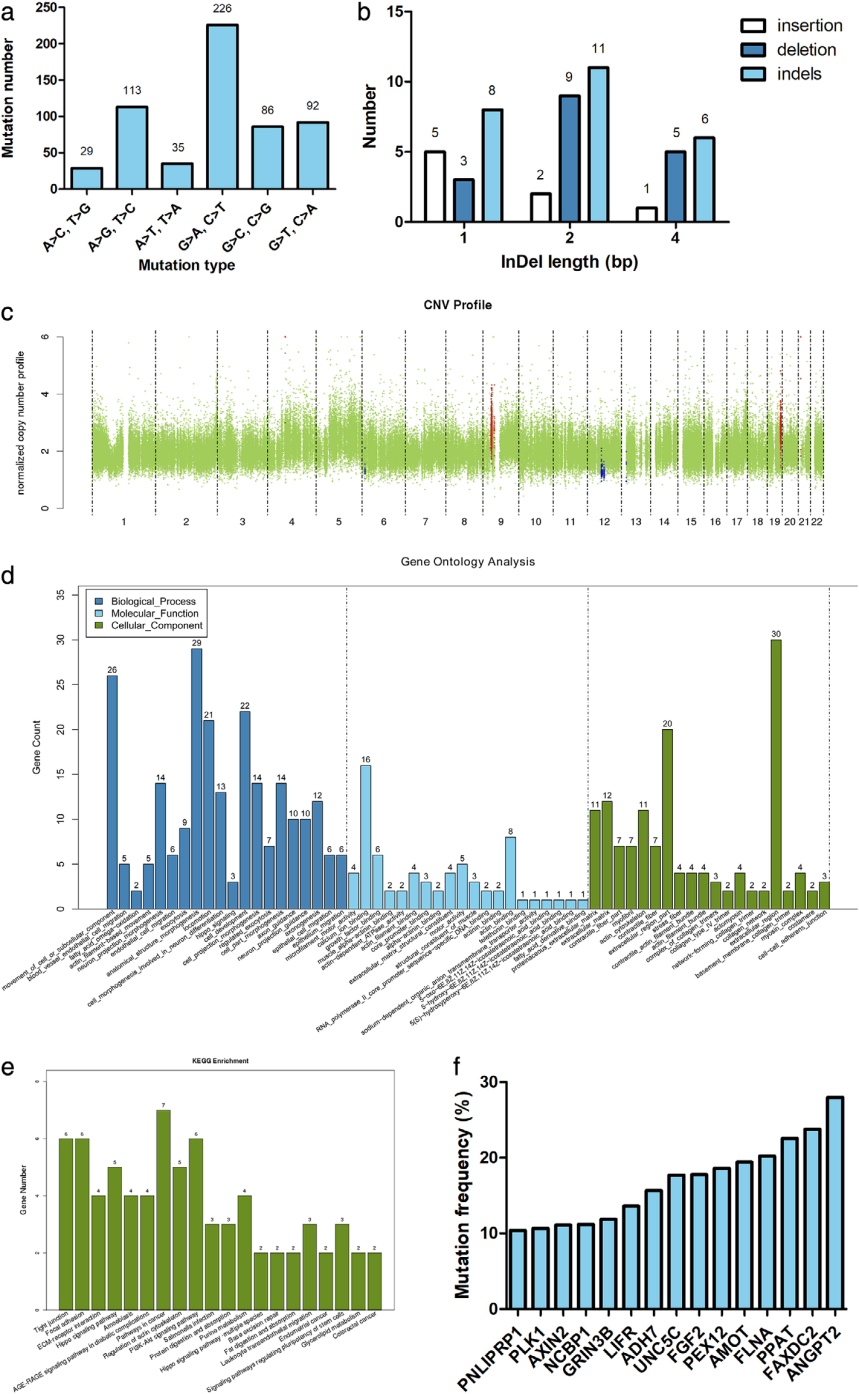
Analysis of the molecular mechanism underlying the resistance of HCC827‐TR cells: **(a)** Types of SNVs in HCC827‐TR cells. **(b)** Distribution of InDeL length in HCC827‐TR cells. **(c)** The CNV results in HCC827‐TR cells. The horizontal axis indicates chromosome numbers, and the vertical axis indicates the change of copy number; the green, blue, and red indicate that the copy number has not changed, was reduced or was increased, respectively. **(d)** The GO enrichment results. **(e)** The KEGG enrichment results. **(f)** The gene mutation frequency is greater than 10%.

The total number of single nucleotide variants (SNV) in the HCC827‐TR cell line was 581, and 228 have been reported in the dbSNP database. Single sample alteration type statistics showed that the highest alterations incidences were G > A, C > T. The least common alterations were A > C and T > G (Fig 3(a)). The results of the InDeL analysis are shown in Figure 3(b). The total number of somatic InDeLs in the HCC827‐TR cells was 38, and 28 have been reported in the dbSNP database; 10 InDeLs were novel; none of the identified InDeLs resulted in a stop codon loss or stop codon gain.

The total number of copy number variation (CNV) in HCC827‐TR cells was 10; they were increased in five regions and reduced in five regions, with a total length of 9 611 185 bp and 1 523 874 bp, respectively. The CNVs were not located in any presently known gene loci that could lead to drug resistance. There were five chromosomes with CNVs, including chromosomes 4, 6, 9, 12, and 21 (Fig 3(c)).

#### GO and KEGG analyses of differentially expressed genes

We next examined the differentially expressed genes between HCC827‐P and HCC827‐TR cells. The distribution of the differentially expressed genes obtained from the NGS in GO enrichment analyses is shown in Figure 3(d). The KEGG pathway enrichment analysis showed that seven differentially expressed genes were enriched in tumor‐related pathways; six genes were enriched in the tight junction, focal adhesion, and PI3‐K‐AKT signaling pathways; five genes were enriched in the Hippo signal pathway and regulation of actin cytoskeleton (Fig 3(e)).

#### Screening of target genes

The specific molecular alterations screened in the drug‐resistant cell lines of the above were compared with the resistance mutations in the GDSC database. A total of 47 genes associated with erlotinib resistance were found in the GDSC database that were also significantly enriched in the KEGG pathway (Table S2). All the differentially expressed genes were associated with SNVs, but not somatic InDeLs or CNVs. Genes with a mutation frequency more than 10% are shown in Figure 3(f). The functional prediction analysis of all 47 genes using Poltphen2, SIFT, Mutation Taster, and CADD all showed that the identified mutations in *FGF2* were deleterious; thus, FGF2 was selected for subsequent experiments.

The results showed that *FGF2* participates in eight signaling pathways, including hsa04010, hsa05205, hsa04015, hsa05200, hsa04014, hsa04151, hsa05218, and hsa04810. The previously reported mutation frequency of *FGF2* was only 0.29%, and most mutations were located in the 150–250 nucleotide region in the COSMIC database. Our results identified a point mutation of G101T in exon 1 on chromosome 4 in HCC827‐TR cell lines, resulting in the substitution of glycine with valine, and the mutation frequency reached 17.8%.

#### Expression of *FGF2* and *FGFR2* and their downstream signal factors

**Figure 4:**
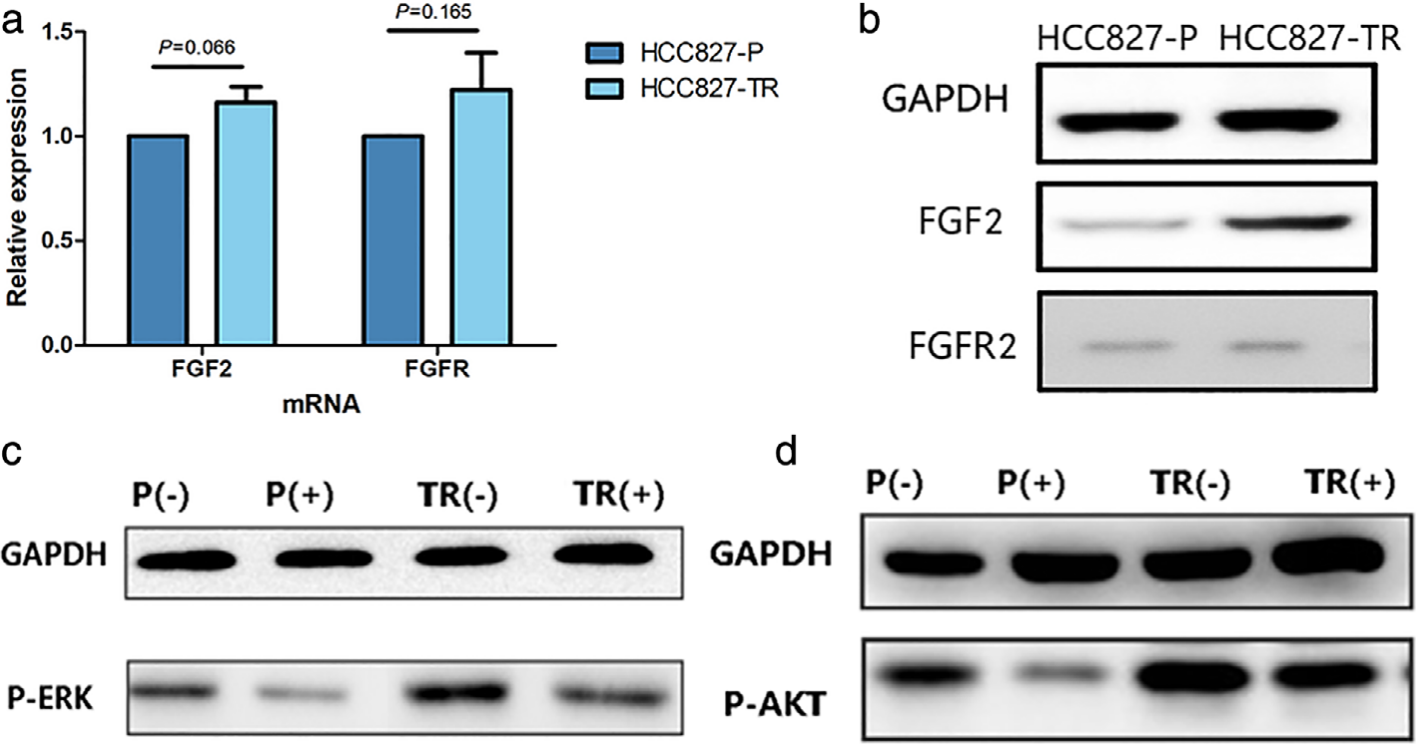
Expression of *FGF2* and *FGFR2* and downstream signal factors. **(a)** RT‐PCR of *FGF2* and *FGFR2* mRNA expression in HCC827‐P and HCC827‐TR cells. **(b)** Western blot analysis of FGF2 and FGFR2 in HCC827‐P and HCC827‐TR cells. **(c)** Western blot analysis of P‐ERK and **(d)** P‐AKT in HCC827‐P and HCC827‐TR cells treated with (+) or without (−) 1 μmol/L erlotinib.

There were no significant differences in the mRNA expression of *FGF2* (*P* = 0.066) and *FGFR2* (*P* = 0.165) in the two cell lines (Fig 4(a)). However, western blot shown that the level of FGF2 protein was significantly higher in HCC827‐TR cells than in the HCC827‐P cells (*P* < 0.05), while the expression levels of FGFR2 in the two cell lines were not significantly different (*P* > 0.05, Fig 4(b)).

The FGF2/FGFR downstream PI3K and mitogen‐activated protein kinase (MAPK) signaling pathways are activated with the phosphorylation of key kinases AKT and ERK, respectively. We compared the levels of P‐AKT and P‐ERK in the two cell lines with or without 1 μmol/L erlotinib. They significantly decreased in HCC827‐P cells treated with 1 μmol/L erlotinib; however, in HCC827‐TR cells, the level of P‐ERK decreased slightly, and P‐AKT showed almost no change after treatment with 1 μmol/L erlotinib compared with the control (Fig 4(c), (d)).

### Targeted inhibition of the FGF2/FGFR signaling pathway

**Figure 5:**
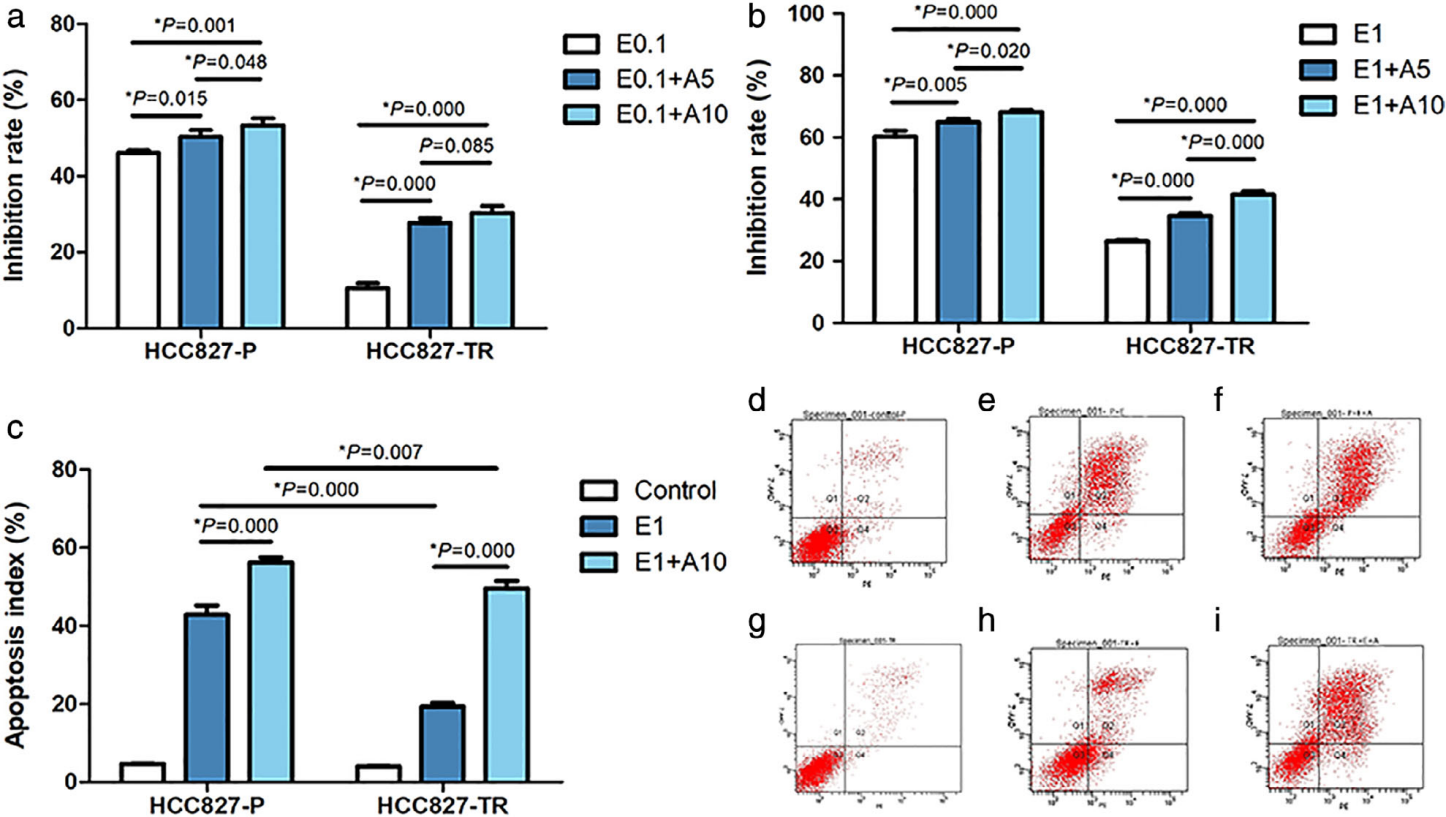
Cell viability and apoptosis assays after targeted inhibition of the FGF2/FGFR signaling pathway: **(a)** MTS assays of HCC827‐P and HCC827‐TR cell lines treated with a low concentration of erlotinib combined with different concentrations of AZD4547. **(b)** MTS assays of HCC827‐P and HCC827‐TR cell lines treated with a high concentration of erlotinib combined with different concentrations of AZD4547. **(c)** The apoptosis index of HCC827‐P and HCC827‐TR cell lines treated with various treatment strategies. Apoptosis scatter plots in HCC827‐P cell lines: **(d)** control, **(e)** E1, **(f)** E1 + A10. Apoptosis scatter plots in HCC827‐TR cell lines: **(g)** control, **(h)** E1, **(i)** E1 + A10. E0.1: erlotinib 0.1 μmol/L; E0.1 + A5: erlotinib 0.1 μmol/L combined with AZD4547 5 μmol/L; E0.1 + A10: erlotinib 0.1 μmol/L combined with AZD4547 10 μmol/L; E1: erlotinib 1 μmol/L; E1 + A5: erlotinib 1 μmol/L combined with AZD4547 5 μmol/L; E1 + A10: erlotinib 1 μmol/L combined with AZD4547 10 μmol/L.

HCC827‐P and HCC827‐TR cell lines were treated with 0.1 (E0.1) or 1 μmol/L (E1) erlotinib combined with 5 μmol/L (A5) or 10 μmol/L (A10) AZD4547 (FGF2/FGFR inhibitor). MTS assay in HCC827‐P cells revealed a significant difference in the inhibition rate between the single drug group and the combined groups (*P* = 0.015, *P* = 0.001, respectively), regardless of the concentration of AZD4547; the results were similar in HCC827‐TR cells (*P* = 0.000, *P* = 0.000). When we compared the two combined treatment groups (E0.1 + A5 and E0.1 + A10), although the inhibition rate showed a statistical difference, the difference was not significant in HCC827‐P cells (*P* = 0.048), and no significant difference was observed in the HCC827‐TR cells (*P* = 0.085) (Fig 5(a)). When the concentration of erlotinib was increased to 1 μmol/L, there were significant differences in the inhibition rates among the E1, E1 + A, and E1 + A10 groups (all *P* < 0.05) in HCC827‐P and HCC827‐TR cell lines (Fig 5(b)). However, under the same drug treatment conditions, the inhibition rate of HCC827‐P cells was still significantly higher than that of HCC827‐TR cells. Although the addition of AZD4547 could restore the sensitivity of HCC827‐TR cell lines to erlotinib, the drug inhibition rate still failed to reach the level observed in the parental cells (Fig 5(a), (b)).

Based on the above MTS results, we selected the E1 and E1 + A10 treatment strategies to treat HCC827‐P and HCC827‐TR cell lines to detect apoptosis. Although the apoptotic level was significantly different between the E1 and E1 + A10 groups in both HCC827‐P (*P* = 0.000) and HCC827‐TR (*P* = 0.000) cells, the rise level of the apoptotic index in HCC827‐TR cells treated with E1 + A10 was significantly higher than that in HCC827‐P cell lines (Fig 5(c)–(i)).

## Discussion

The mechanism of EGFR‐TKI resistance has been defined at around 60%–70%, classified into the secondary mutations in *EGFR*, bypass or alternative activation, and histological or phenotypic transformation. To examine potential novel resistance mechanisms, we first constructed an EGFR‐TKI resistant model in vitro using the primary *EGFR* sensitive mutation (E746–A750 deletion) HCC827‐P cell line and established HCC827‐TR resistant cells. MTS assays revealed that IC_50_ of HCC827‐P cells was about 0.1 μmol/L, while that of HCC827‐TR cells was at least 10 μmol/L, indicating that its resistance was 100 times higher than that of the primary cells. These results confirmed that the HCC827‐TR cell line was an EGFR‐TKI resistant cell line.

Sanger sequencing was used to examine secondary mutations in *EGFR* at the DNA level; the results revealed that both HCC827‐TR and HCC827‐P cell lines did not contain the T790M mutation. Therefore, the mechanism of resistance was not related to the secondary mutations. Meanwhile, the expressions of proto‐oncogene *MET* and anti‐oncogene *PTEN* in two cell lines were not significantly different.

NGS was used to examine whole exons of the two cell lines, and after a series of bioinformatics analyses, we selected *FGF2* for subsequent studies. Previous studies have shown that FGF2 can promote the growth of malignant tumors via autocrine and paracrine pathways and that it plays an important role in tumor migration.^20^ Berger *et al*.^21^ first confirmed the existence of an FGF2‐FGFR autocrine ring in NSCLC, and this abnormal activation was associated with poor prognosis. Ware *et al*.^22^ demonstrated that EGFR‐TKI‐induced FGFR2 and FGFR3 are capable of mediating FGF2 and FGF7‐stimulated ERK activation, highlight EGFR‐TKI‐induced FGFR2 and FGFR3 signaling as a rapid mechanism of acquired resistance.

The point mutation of G101T in exon 1 in *FGF2* resulted in substitution of glycine by valine. We did not detect any difference in the mRNA expression of FGF2 in the two cell lines; however, FGF2 protein expression was significantly higher in HCC827‐TR cell lines compared with that in the parental cells. Moreover, the expression of FGFR showed no significant differences between the two cells, both in the mRNA and protein levels. We believe that the point mutation in *FGF2* may result in the conformational changes or altered binding sites of the translated protein, resulting in hindered degradation of FGF2. High levels of FGF2 binding with FGFR can activate the signaling pathway and induce cell proliferation and resistance. In addition, the expressions of the P‐AKT and P‐ERK were significantly downregulated in response to 1 μmol/L erlotinib in HCC827‐P cells, while the continuous activation of P‐AKT and P‐ERK was still present in the HCC827‐TR cell line after the addition of erlotinib. This suggests that the FGF2‐FGFR continuous activation of the downstream network signal pathway through bypass and the growth of the tumor cells is not restricted by the inhibitory effect of EGFR‐TKI. However, this is only a hypothesis, and further experiments are needed to verify this possibility.

The fibroblast growth factor receptor pathway plays a key role in signal transduction in lung cancer; it can activate multiple signal transduction pathways, including rat sarcoma (RAS) kinase and MAPK, which in addition to performing proliferative functions, controls cellular processes, such as cell cycle progression, migration, metabolism, and differentiation.^23^ In the last few years, several potent and specific inhibitors of FGFR have been introduced into the clinic, such as FGFR1/2/3 inhibitors (AZD4547, dovitinib/TKI258), selective FGFR4 inhibitors (BLU9931), and pan‐FGFR inhibitors (erdafitinib/JNJ‐493, LY2874455). According to the http://clinicaltrials.gov website, 26 FGFR‐targeted agents are currently under investigation in clinical trials, of which 13 agents are being tested in phase I trials, 10 agents are being investigated in phase II trials, AZD4547 and erdafitinib are in phase III, although no drugs have been approved for lung cancer, some studies have shown positive results.

AZD‐4547 is a highly selective FGFR inhibitor; a patient‐derived xenograft model of FGFR1‐amplified lung cancer was highly sensitive to AZD4547 as single agent.^24^ Our experimental results notably indicate that AZD‐4547 combined with erlotinib significantly restored the sensitivity of HCC827‐TR cells to the EGFR‐TKI both in low‐ and high‐concentration groups. The cell inhibition rate of HCC827‐P cells in response to the combination treatment also increased slightly compared to that with erlotinib alone; however, the difference was not statistically significant. These results indicated that the drug resistance of HCC827‐TR cells to the EGFR‐TKI was reversed by the targeted inhibition of the FGF2/FGFR signaling pathway and confirmed that *FGF2* mutation is an important mechanism of resistance in HCC827‐TR cells. Our apoptosis results also confirmed this conclusion.

In conclusion, we successfully constructed an erlotinib‐resistant cell line HCC827‐TR and identified EGFR‐TKI resistance mechanism involving FGF2 gene mutation. It is noteworthy that the targeted inhibition of the FGF2/FGFR signaling pathway could effectively restore the sensitivity of the resistant cells to erlotinib. These results not only expand our understanding of the molecular mechanisms underlying drug resistance in NSCLC cancers, but also suggest a novel treatment strategy for EGFR‐TKI resistant NSCLC patients.

## Disclosure

No authors have financial or other contractual agreements that might cause conflicts of interest.

## Supporting information


**Table S1** Primers used for RT‐PCR.

**Table S2** The mutation frequency of differentiated genes in HCC827‐TR cell lines.
