# Supplementary material for: Molecular mechanism of acquired drug resistance in the EGFR‐TKI resistant cell line HCC827‐TR
Source: Thorac Cancer. 2020 Mar 12;11(5):1129–38. doi: 10.1111/1759-7714.13342 (PMC7180561; doi:10.1111/1759-7714.13342)
Supplement: Supplementary file 1 — Table S1 Primers used for RT‐PCR. [file TCA-11-1129-s001.docx]

**Table S1 Primers used for RT-PCR**

| **Gene** | **Oligonucleotide sequence (5’-3’)** |
| --- | --- |
| GAPDH  Forward primer  Reverse primer | GGAGTCAACGGATTTGGTCG  CTTGATTTTGGAGGGATCTCG |
| MET  Forward primer  Reverse primer | CCATCCAGTGTCTCCAGAAGTG  TTCCCAGTGATAACCAGTGTGTAG |
| PTEN  Forward primer  Reverse primer | GCCTCATCGAGGAAAAACAGG  GTCTCGTGTCCAACGGGTC |
| EGFR  Forward primer  Reverse primer | CTCCCTCCAGGAAGCCTACGTGAT  TTTGCGATCTGCACACACCA |
| FGF2  Forward primer  Reverse primer | CGGCTGTACTGCAAAAACGG  GATGTGAGGGTCGCTCTTCTCC |
| FGFR2  Forward primer  Reverse primer | GAAAGATGCCGCCGTGATCAG  CACCACCATGCAGGCGATTAAG |
| Bax  Forward primer  Reverse primer | GGGTGGTTGGGTGAGACTC  AGACACGTAAGGAAAACGCATTA |
| Bcl-2  Forward primer  Reverse primer | GAACTGGGGGAGGATTGTGG  CCGGTTCAGGTACTCAGTCA |
| CCND1  Forward primer  Reverse primer | GAACAAACAGATCATCCGCAAAC  GCGGTAGTAGGACAGGAAGTTG |
| Ras  Forward primer  Reverse primer | CGATGGAACTTCGACTTTGTCA  GCACAAGGGTACAAGACAGTG |
