# Supplementary material for: Molecular mechanism of acquired drug resistance in the EGFR‐TKI resistant cell line HCC827‐TR
Source: Thorac Cancer. 2020 Mar 12;11(5):1129–38. doi: 10.1111/1759-7714.13342 (PMC7180561; doi:10.1111/1759-7714.13342)
Supplement: Supplementary file 2 — Table S2 The mutation frequency of differentiated genes in HCC827‐TR cell lines. [file TCA-11-1129-s002.docx]

**Table S2 The mutation frequency of differentiated genes in HCC827-TR cell lines**

| Gene | Chromosome | Location | Original base | Mutational base | Mutation frequency (%) |
| --- | --- | --- | --- | --- | --- |
| PTGFR | chr1 | 79002208 | C | T | 1.63 |
| PIGM | chr1 | 160000622 | G | A | 4.44 |
| LTBP1 | chr2 | 33487810 | G | T | 4.09 |
| CTNNA2 | chr2 | 80874809 | A | G | 6.47 |
| TTN | chr2 | 179600337 | C | G | 8.9 |
| COL4A4 | chr2 | 227983389 | C | G | 7.1 |
| HYAL3 | chr3 | 50330823 | G | A | 4.7 |
| NUDT16 | chr3 | 131101044 | C | G | 3.521 |
| HTR3E | chr3 | 183818242 | C | T | 6.25 |
| POLR2H | chr3 | 184081299 | G | C | 3.305 |
| PPAT | chr4 | 57273847 | C | G | 22.58 |
| UNC5C | chr4 | 96091414 | C | T | 17.7 |
| ADH7 | chr4 | 100349669 | C | G | 15.7 |
| FGF2 | chr4 | 123748031 | G | T | 17.8 |
| LIFR | chr5 | 38499675 | C | A | 13.6 |
| FAXDC2 | chr5 | 154203121 | C | T | 23.8 |
| HIST1H4A | chr6 | 26022006 | G | A | 1.626 |
| MYL7 | chr7 | 44179978 | C | T | 1.96 |
| ABCA13 | chr7 | 48313099 | A | C | 5.26 |
| COL1A2 | chr7 | 94058599 | C | T | 4.76 |
| EPHB4 | chr7 | 100417286 | C | T | 3.07 |
| ANGPT2 | chr8 | 6377495 | C | A | 28 |
| NCBP1 | chr9 | 100421016 | G | T | 11.2 |
| NOTCH1 | chr9 | 139399868 | G | C | 1.89 |
| VCL | chr10 | 75863631 | T | G | 9.86 |
| PNLIPRP1 | chr10 | 118351997 | A | C | 10.4 |
| EIF3A | chr10 | 120824949 | G | C | 3.00 |
| PNPLA2 | chr11 | 824433 | C | G | 8.1 |
| TEAD4 | chr12 | 3103936 | G | A | 5.52 |
| AGAP2 | chr12 | 58129187 | A | G | 1.68 |
| UPF3A | chr13 | 115047302 | A | G | 9.09 |
| POLE2 | chr14 | 50133101 | T | C | 6.93 |
| PLK1 | chr16 | 23701181 | G | A | 10.7 |
| MYH13 | chr17 | 10223672 | T | A | 3.92 |
| PEX12 | chr17 | 33903006 | G | C | 18.6 |
| STAT5A | chr17 | 40451789 | C | A | 2.5 |
| CSH2 | chr17 | 61950577 | G | A | 1.54 |
| AXIN2 | chr17 | 63537630 | C | A | 11.1 |
| GRIN3B | chr19 | 1003718 | C | T | 11.86 |
| MYH14 | chr19 | 50795620 | G | C | 5.43 |
| LILRA1 | chr19 | 55106288 | A | C | 2.54 |
| GP6 | chr19 | 55538987 | A | G | 9.93 |
| CDH4 | chr20 | 60470012 | G | T | 2.89 |
| MYH9 | chr22 | 36684345 | C | T | 4.71 |
| COL4A5 | chrX | 107938108 | C | T | 3.84 |
| AMOT | chrX | 112022504 | C | A | 19.44 |
| FLNA | chrX | 153581521 | T | C | 20.2 |
